# Living with advanced cancer: Rich Pictures as a means for health care providers to explore the experiences of advanced cancer patients

**DOI:** 10.1002/cam4.2342

**Published:** 2019-07-06

**Authors:** Zarah M. Bood, Michael Scherer‐Rath, Mirjam A. G. Sprangers, Liesbeth Timmermans, Ellen van Wolde, Sayra M. Cristancho, Fenna Heyning, Silvia Russel, Hanneke W. M. van Laarhoven, Esther Helmich

**Affiliations:** ^1^ Department of Medical Oncology, Cancer Center Amsterdam Amsterdam University Medical Centers, University of Amsterdam Amsterdam The Netherlands; ^2^ Faculty of Philosophy, Theology, and Religious Studies Radboud University–Nijmegen Nijmegen The Netherlands; ^3^ Department of Medical Psychology Amsterdam University Medical Centers, University of Amsterdam Amsterdam The Netherlands; ^4^ Department of Primary and Community Care Radboud University Medical Centre Nijmegen The Netherlands; ^5^ Centre for Education Research & Innovation and Department of Surgery, Health Sciences Addition, Schulich School of Medicine and Dentistry Western University Ontario Canada; ^6^ Association of Topclinical Hospitals STZ Utrecht the Netherlands; ^7^ Independent Artist, Researcher Amsterdam the Netherlands; ^8^ Center for Education Development and Research in Health Professions University Medical Center Groningen, University of Groningen Groningen the Netherlands

**Keywords:** advanced cancer, health care professionals, patients’ experiences, Rich Pictures, visual tool

## Abstract

**Background:**

To provide holistic care to patients with advanced cancer, health care professionals need to gain insight in patients’ experiences across the different domains of health. However, describing such complex experiences verbally may be difficult for patients. The use of a visual tool, such as Rich Pictures (RPs) could be helpful. We explore the use of RPs to gain insight in the experiences of patients with advanced cancer.

**Methods:**

Eighteen patients with advanced cancer were asked to draw a RP expressing how they experienced living with cancer, followed by a semi‐structured interview. Qualitative content analysis, including the examination of all elements in the drawings and their interrelationships, was used to analyze the RPs, which was further informed by the interviews.

**Results:**

The RPs clearly showed what was most important to an individual patient and made relations between elements visible at a glance. Themes identified included: medical aspects, the experience of loss, feelings related to loss, support from others and meaningful activities, and integration of cancer in one's life. The added value of RPs lies in the ability to represent these themes in one single snapshot.

**Conclusions:**

RPs allow for a complementary view on the experiences of advanced cancer patients, as they show and relate different aspects of patients’ lives. A RP can provide health care professionals a visual summary of the experiences of a patient. For patients, telling their story to health care professionals might be facilitated when using RPs.

## 1 INTRODUCTION

The diagnosis of advanced, incurable cancer is likely to have a profound impact on people.^1^, ^2^, ^3^, ^4^, ^5^ The limited lifetime left may directly conflict with the goals people have in life, and, as such, may entail an experience of contingency.^3^, ^4^ Contingency refers to the randomness of life, that everything could have been different.^3^, ^4^, ^5^, ^6^ When life goals are jeopardized, questions like “why me” and “why now” may arise.^1^, ^4^, ^5^, ^7^ Patients need to make meaning of their life with cancer and incorporate diagnosis and prognosis in their life story.^5^ Integrating advanced cancer in one's narrative of life, however, is challenging, and feelings of hopelessness, depression, and even the desire for a hastened death were found to be common among patients with advanced cancer.^1^, ^2^ Therefore, care for patients with advanced cancer needs to address psychosocial, spiritual and existential health in addition to physical health.^3^, ^7^, ^8^, ^9^, ^10^ Despite improvements in cancer care in developed countries, these nonphysical domains remain largely unattended.^10^, ^11^, ^12^

The experience of contingency can be conceptualized as an interpretation crisis, and people may not be able to find words to express what is happening to them, that is, words may be insufficient in capturing intense experiences.^4^, ^5^, ^6^ Thus, the use of questionnaires or in‐depth interviews can only uncover part of patients’ experiences.^10^, ^13^ A different approach invites patients to tell their story through visuals, in addition to spoken language.^14^, ^15^, ^16^, ^17^, ^18^ Various visual tools have been used among cancer patients in developed countries to evoke these visual narratives, such as drawings, paintings, and comics.^18^, ^19^, ^20^ Furthermore, visual tools have been used in community work in for example Sub‐Saharan countries.^21^ The combination of verbal and visual tools was found to be helpful in unravelling experiences of patients with cancer.^18^, ^19^, ^20^ However, previously used tools only focussed on one specific experience, such as physical pain or anxiety.^18^, ^19^, ^20^

A visual tool that could provide a more comprehensive view of the experiences of patients with cancer is the Rich Picture (RP).^14^, ^16^, ^17^, ^22^, ^23^ A RP is a drawing that someone creates about their experience, including all the people, materials, processes, interactions, and feelings that contribute to the experience.^14^, ^17^, ^23^ RPs originate from systems engineering and were originally used to explore complexity, but RPs have recently also been used in, for example, medical education research with surgical experts and with medical trainees, inviting participants to draw a complex and challenging situation in their work.^14^, ^16^ RPs were found to aid understanding, dialogue and the sharing of experiences.^14^, ^16^, ^17^, ^24^ To the best of our knowledge, RPs have not yet been applied to capture the experiences of patients. Our aim was to explore the use of RPs and subsequent interviews as a method to gain insight in the experiences of patients with advanced cancer in an academic medical centre in the Netherlands.

## 2 METHODS

### 2.1 Study design and participants

We adopted an interpretive qualitative approach that was largely guided by the principles of constructivist grounded theory, such as purposive sampling and constant comparison.^25^, ^26^ An iterative approach to data collection and data analysis was taken, that is, data analysis started alongside data collection to inform subsequent sampling and data collection.

Patients were recruited from the Department of Medical Oncology of the Amsterdam University Medical Centers, Location AMC, the Netherlands. We asked patients above the age of 18 with a diagnosis of advanced, incurable cancer, receiving palliative treatment and/or best supportive care, to participate. Patients with all types of solid tumors who were sufficiently fit to participate, were eligible. Patients were approached during appointments at the hospital by their attending oncologist. We aimed to include approximately 15 patients, a sufficiently large sample size to obtain meaningful findings with a grounded theory research design.^25^

### 2.2 Data collection

The first author (ZB), a PhD student with a research masters in global (mental) health, conducted all the interviews. Three pilot interviews were carried out to train ZB in RP interviewing and were discussed with an experienced RP researcher (EH). We asked patients to make an RP about their experience of living with advanced cancer, followed by a semi‐structured interview. At the start of each RP session, we used a published example of an RP as an example to show patients which icons and symbols could be included and how it might look when completed.^22^ This specific example was also used in previous RP research.^14^, ^16^ Patients were provided with an A1 paper sheet, colored pencils and markers, and were given the time they needed to draw the RP, with a maximum of 30 minutes. This maximum was chosen to prevent overburdening patients and for the reason that in clinical practice more than 30 minutes is expected not to be feasible because of time restraints. When the drawing was finished, patient and interviewer engaged in an interview about the RP. The interviewer started with the open question “Can you explain to me what you have drawn?”, and then asked more specific questions about elements of the RP (eg about the colors, shapes, specific elements, and relationships between them). The interviewer aimed to understand each element of the RP, the meaning behind them and the reason they were drawn. In total, the RP session would take around 1 hour.

### 2.3 Data analysis

Interviews were transcribed verbatim and were used to support the analysis of the RPs. To analyze the combined “Rich Picture/interview” data, 2 main strategies were used: RP viewing sessions and gallery walks. During RP viewing sessions one single RP was discussed in detail for an hour by 6‐8 researchers who had experience with RP research. We analyzed the RPs using content analysis.^27^, ^28^ An analytical framework, based on the work of Carney and adopted by Bell, Berg, and Morse was used to guide the analysis.^24^, ^29^ The aim of these sessions was to obtain multiple perspectives on the content of the RP and explore different ways of seeing. Analysis included the examination of all elements of the drawings, such as facial expressions (eg smiling or crying) and body language (eg holding hands) of figures, use of color, arrows, thought and speech bubbles, placement and interrelatedness of elements, size of elements, and the use of metaphors and symbols.

To gain insight in patterns, differences, and similarities across the whole set of RPs, we organized 3 gallery walks in which all RPs were hung in a room in random order. All attendees would walk around the room to get a first impression of the RPs, and would then sit down to discuss the RPs together. In order to allow for multiple perspectives to enrich the interpretation, these gallery walks were held with different participants. The first 2 gallery walks included researchers with experience in RP research and members of the research team respectively, building on backgrounds as diverse as medicine, psychology, theology, arts, systems engineering and qualitative research. To validate our interpretations, we held the third gallery walk with 4 (former) patients with cancer. These patients, 2 males and 2 females, were all treated for stomach or esophageal cancer and were currently free of disease.

Finally, the first researcher combined the interpretations from the RP viewing sessions and gallery walks into a table in Word, to which we applied open coding to the RPs and the corresponding interview text to create initial codes. The first researcher subsequently clustered codes into themes. Evolving interpretations were discussed and refined in weekly meetings between ZB and EH and presented to the research team 3 times during the analysis process. To interpret the findings in the context of experiences of contingency, the analysis was further informed by a theoretical model that is based on previous research on contingency of Hartog et al^5^

### 2.4 Ethical considerations

The Medical Ethics Review Committee of the Academic Medical Centre stated that no ethical approval was needed for the study. Confidentially of patients was ensured and all collected data were coded and stored in a protected database in the hospital. Prior to participation, all patients were informed about the potential risk of emotional distress and their right to withdraw from the study at any moment. We obtained written informed consent from each patient.

## 3 RESULTS

In total, 18 patients were included in the study, of which 11 were female. Patients’ age ranged from 31 to 81 (mean age 62). Patients had esophageal, stomach, pancreas, colon, or ovary cancer. The sample included patients who were diagnosed with advanced cancer just 2 months before the interviews, while others had lived with advanced cancer for 1‐4 years. Almost all patients had a partner. The characteristics of each patient can be found in Table A1.

For the recognition of the different elements in the RPs, the input of patients during the gallery walk was vital. Patients participating in the gallery walk recognized many elements of the RPs that were not readily identified by researchers and health professionals, such as specific physical sensations and emotions. The main findings of the gallery walks were that a great loss and many intense emotions were depicted, and that relationships between people appeared as a central theme in the RPs. Additionally, the experience of contingency and the way patients dealt with it, in order to restructure their life story, were represented in the RPs.

Based on the RPs and interviews, we identified 5 themes (Table 1), which together formed an overarching narrative around contingency and show how patients shape and transform their individual accounts of their experiences. Patients’ accounts often started with medical aspects and the physical experience of having cancer, which was followed by the experience of loss, feelings around the loss, being supported by others and through meaningful activities, and integrating the cancer in a new life story. We will illustrate how those themes interrelate by presenting 3 individual patient stories, that serve as exemplar cases representative of our main findings. Fictional names are used, but RPs and quotes are numbered according to process of anonymization.

**Table 1:** The five themes with exemplary pieces of RPs and related quotes

Theme	Part of a RP	Quote
Medical aspects and the physical experience of having cancer	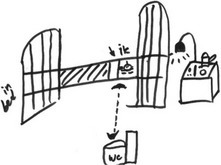	I drew myself, here, that is me. Me in bed. […] And very close to the toilet (WC), because now that is of course the biggest disaster, that you have to go to the toilet all the time and stomach ache and everything. – P14
The experience of loss	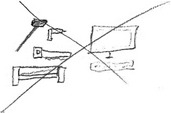	Well, that I don't work, I am no longer able to. – P7
Feelings around the loss	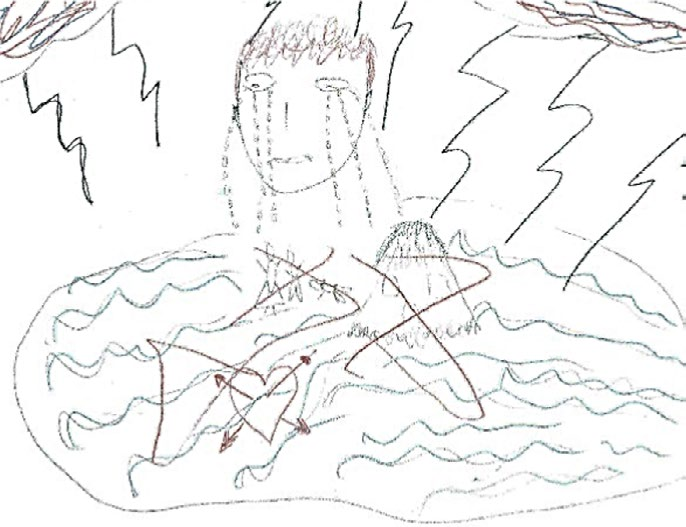	And now I feel more like… like from all angles a dagger has been stabbed through my heart [cries]. […] Now my tears just fill a pool you know. I don't want to do it regularly in front of my family, but when I am alone it feels like the tears won't stop coming. – P5
Being supported by others and through meaningful activities	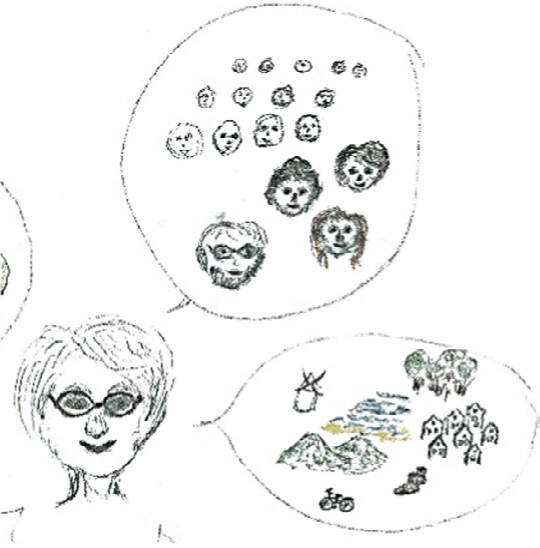	Since I have cancer I have received an incredible amount of support from my husband and my sister and her daughters, my cousins. But also from colleagues, from friends, and from family, well incredibly much, I never expected that to be honest. […] Besides, there are holiday and leisure activities […] – P3
Integrating the cancer in a new life story	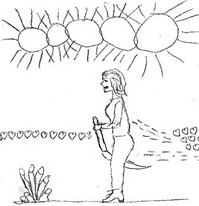	This is me with a scythe in my hands. This is the cancer, which I am fighting. Suns for positivity. Because I am trying really hard to stay really positive. – P8

Medical aspects and the physical experience of having cancer were for example drawn by patients lying in bed. The experience of loss was often illustrated by crosses through the lost elements, such as work. Feelings around the loss of these elements included sadness and anger. However, patients were supported by family and friends, and tried to engage in meaningful activities. How patients related to the cancer, and whether they were able to integrate the disease in their life, was made visible, for instance by depicting how patients fought the cancer and tried to stay positive.

### 3.1 Frank

**Figure 1:**
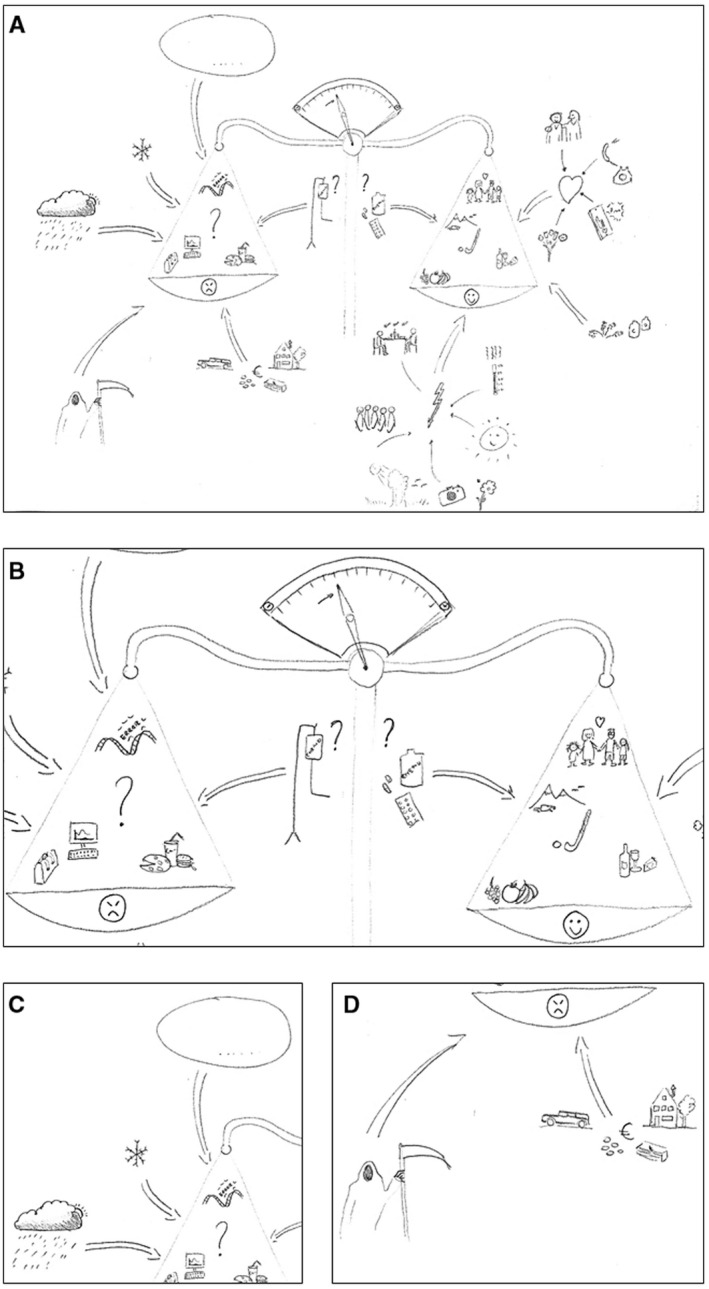
Rich Picture drawn by patient P6. (A) The Rich Picture (B) A scale with a negative anchor on the left and a positive anchor on the right, in which factors of the patient's life are drawn. The negative anchor includes the question about what caused the cancer: work related stress (suitcase and computer), unhealthy eating habits, or a error in DNA. The positive anchor includes family, doing sports, going on holiday, eating healthy, and in general enjoying life. Chemotherapy is drawn in the middle of the scale with questions marks, representing the consideration whether the therapy is negative or positive. (C) Factors that affect the negative anchor of the scale: feeling useless (empty ballon), cold weather that increases neuropathy (snowflake), and feeling sad (rain cloud). (D) Thoughts about death and worries about the financial status of the family have a negative effect on the life of the patient

Frank was a 49‐year‐old male with stomach cancer. Two months before the interview he was diagnosed with advanced cancer and he was now receiving chemotherapy. When asked to make a drawing that represented his experience with living with advanced cancer, he drew a scale with a negative and a positive anchor, symbolizing his life (Figure 1A). The anchors were filled up with the most important elements of his life. In the interview, he explained:

“Well as you can see, it's a big scale for me, which symbolizes life and well, for me it is not in balance. He tipped a bit to… well, I have the positive and the negative side and of course because of the cancer he tipped to the negative side.” – P6

Around the scale, Frank drew other factors that affected the anchors. In this way he identified the main factors and clarified whether they positively or negatively impacted his life. In response to the contingent experience of getting cancer, he considered as the most important medical aspects, the factors that might have caused the cancer and the underlying question whether the disease was his own fault (Figure 1B). These included his demanding work schedule and related stress, unhealthy eating habits, and a possible error in his DNA. The experience of loss was closely related to some of the physical aspects of having cancer, such as fatigue and neuropathy (Figure 1C), and resulted in him not being able to work, causing him to feel useless (Figure 1C). As a consequence of the experience of loss, he depicted feelings of sadness (Figure 1C), worries about his financial status (Figure 1D), and thoughts about approaching death (Figure 1D). On the positive side of the scale, he depicted his family, doing sports, going on holiday, eating healthy, and in general enjoying life (Figure 1B).

Despite the fact that he drew more elements around the positive anchor, a pointer on top of the scale indicated that his experience was more heading toward the negative side of the scale (Figure 1B). Frank explained that the cancer limited him in doing the positive things. However, the pointer was now going in the other direction, because he was trying to regain a positive life balance by focusing on the positive factors that he was still able to do, as a means of integrating the cancer in his life story.

“Yeah, you are not able to do much. That is why I indeed like to meet up with people and also to keep playing sports if I am physically able to […]. So I regularly meet up with people of whom I think, yes I like to see him or her. Yes I find that important now. Something that you always used to postpone like, well I am busy, I don't have time for this, it will come another time, I now think, no I will just do it right away.” – P6

In response to the tragic of losing his health and ultimately his life, Frank described how he shifted priorities, which can be interpreted as a transformation of his life story.

### 3.2 Leo

**Figure 2:**
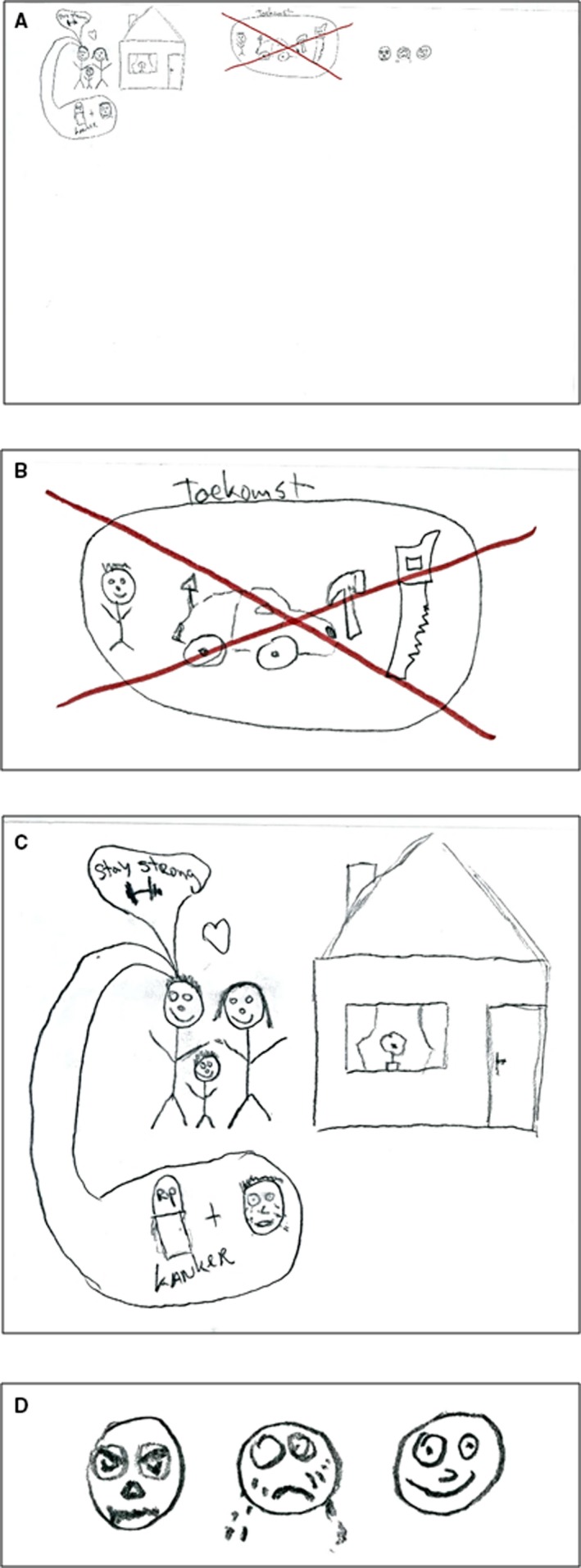
Rich Picture drawn by patient P2. (A) The Rich Picture (B) A red cross is drawn through the future of the patient, which included his son, his dream car, and his career as carpenter. (C) The patient and his family standing next to their house. The patient is thinking about his approaching death because of the cancer, but is trying to stay strong for his family. (D) The patient has mixed emotions, he is often angry and sad, but sometimes he feels happy when spending time with his family. Translations: *Toekomst = Future; *Kanker = cancer

Leo, was a 31‐year‐old male with advanced esophageal cancer. He had lived with the diagnosis for a little more than 2 years and had recently decided to stop chemotherapy. In his drawing, he did not depict any medical aspects, but focused on the experience and feelings related to loss, indicated by a big red cross through his future (Figure 2A,C). The loss of future made him not only sad, but also angry (Figure 2D). He was angry about losing his career prospects, which made him feel useless, and about getting cancer at such a young age. Despite the central experience being one of loss, he could still feel happy when spending time with his family. Leo was clearly struggling with the integration of the cancer and approaching death into his life story, feeling sad, but on the other hand wanting to stay strong for his family (Figure 2B).

The experience of loss that is central to this RP was also strongly conveyed by the large empty space that is part of the RP (Figure 1A). Only when drawing, Leo realized how small his world had become, as illustrated by the following quote:

“It's also abrupt you know, it's suddenly from everything to nothing. Well, yeah, you can actually see that in my drawing as well. And that shocks me to be honest. […] When you are telling someone it still seems quite something, but now that I see it on paper, the only thing I actually still have are my family and my house. The rest is just gone. […] I think that this shows that in reality it is less glamorous than when I would just tell someone.” – P2

### 3.3 Rachel

**Figure 3:**
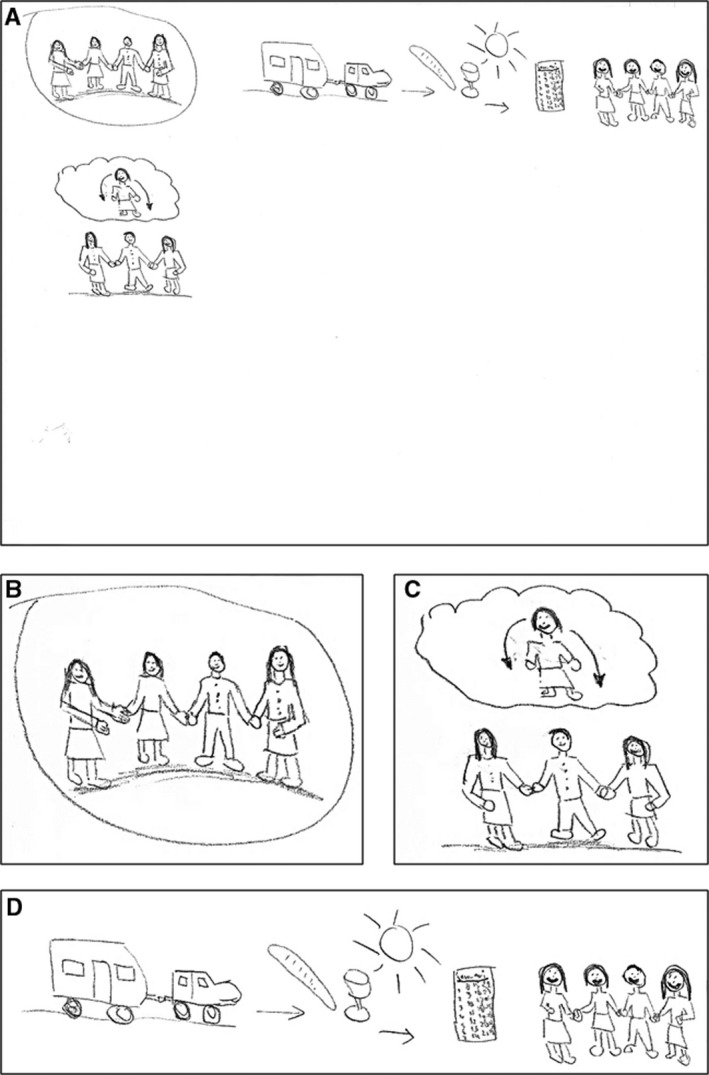
Rich Picture drawn by patient P9. (A) The Rich Picture (B) The patient and her family holding hands to show how close and supportive they are. (C) The patient after her death, looking down on her family with a smile. (D) The hope that the patient is still able to go on holiday and enjoy life together with her family. A calendar with January writing on it indicating that the patient hoped to live until at least that month

Rachel, a 63‐year‐old female, lived with advanced esophageal cancer for a little more than 2 years. She drew no medical aspects and the loss she experienced was not directly clear from the RP (Figure 3A). Instead, she focused on her family, how they supported each other, and how she hoped to spend as much time with them as possible (Figure 3B,D). After the loss of previous life goals, her wish was now that her family would support each other and enjoy their life, even after she has gone (Figure 3C): *“I am looking happily at them, at their happy faces, that they may be happy as well, not because I am gone, but because they are together, the 3 of them, that they can support each other. That is my goal actually.”*

When asked to reflect on the experience of making a drawing in comparison to talking without having to draw first, Rachel said:

“Yes it is clear that this is different, because now you are talking about what you have drawn, about your feelings about what you have drawn. And otherwise this may have been a completely different conversation, well… more superficial, let's call it that way, this is deeper, this goes deeper into your feelings. That is how I feel it at least, because you are drawing what is going on in your mind.” – P9

## 4 DISCUSSION

This is the first study using RPs in advanced cancer patients. RPs helped patients to tell their story and talk about what is most important to them and what other factors, aside from the disease, play a role in their life. The most dominant themes shared through the RPs and interviews were the experience and feelings around loss, and the importance of social relationships. Social relationships provided support and comfort, but also caused sadness, because patients realized they had to leave their loved ones behind. The themes found in our study correspond with findings of previous studies that have used questionnaires and in‐depth interviews.^9^, ^30^, ^31^, ^32^, ^33^ Indeed, rather than providing new themes, the added value of RPs lies in the ability to represent the themes in one single snapshot. It provides a complementary view on the experiences of patients and gives insight into the relevant themes and how these interact. Furthermore, creating an RP can be considered an introductory step in narrative meaning making itself, as drawing one's story might stimulate reflection and meaning making in the interpretation crisis resulting from an incurable cancer diagnosis.^5^, ^10^, ^16^ While RPs touch upon the aspects of art‐based therapy, art‐based therapies target the integration of a disease in their life to develop a new life story.^34^ Slightly countering this, RPs are about helping patients to construct and tell their current life story, not create a new one.^21^ RPs can lay the foundation for art‐based therapy by gaining insight into the current life story of the patients before developing a new life story.

Some limitations of our study need to be acknowledged. First, engaging in a RP session of approximately an hour may be too burdensome and therefore less feasible for patients who are in a more advanced disease stage. Additionally, not all patients will feel comfortable with expressing themselves visually. Related to the study design, only one interviewer conducted all the interviews. The choice of interviewer may influence the data collection as patients are more likely to open up when they feel comfortable with the interviewer. Hence, data could have been different with another interviewer. Also, the stakeholders participating in data analysis may have shaped the interpretation of the data. We therefore included a diversity of relevant stakeholders.

These limitations are all related to the qualitative approach in the use of RPs. However, despite the disadvantages of such a qualitative measure, in our opinion, the large advantage of RPs, that is, being able to provide a visual summary of patients’ experiences, outweighs these disadvantages. Also, we acknowledge that our findings are culturally bound and relate to a Dutch way of sharing experiences. How comfortable patients are with this method depends on their cultural background. To examine whether RPs can be used in patients with different backgrounds, RP research should be conducted in other cultural groups and countries.

The clinical implications of RP interviews are noteworthy. By using RPs, it is possible to literally see what the experiences and feelings of an individual patient are. Thus, RPs may help health professionals to gain insight in the perspective of the patient. For patients, telling their story to health care professionals might be facilitated if they could refer to the visuals used in the RP. Thus, we envision a practice where patients are stimulated to make an RP at home or in a meeting with a palliative care counsellor, and then bring this RP to an appointment with their attending clinician. Alternatively, given the finding that patients attending the gallery walk easily recognized the expressed elements of the RPs, a collection of symbols and metaphors drawn in RPs could be made and used when talking to patients. Health care professionals could ask patients which symbols and metaphors they recognize from their own experiences and which they find most important, creating a low‐threshold starting point for patients to talk about their concerns.

## CONFLICT OF INTEREST

None.

## AUTHOR CONTRIBUTIONS

Zarah M. Bood: patient recruitment, data collection, data analysis and data interpretation, manuscript writing, manuscript review. Michael Scherer‐Rath: data analysis and data interpretation, manuscript review. Mirjam AG Sprangers: data analysis and data interpretation, manuscript review. Liesbeth Timmermans: data analysis and data interpretation, manuscript review. Ellen van Wolde: data analysis and data interpretation, manuscript review. Sayra M. Cristancho: data analysis and data interpretation, manuscript review. Fenna Heyning: data analysis and data interpretation, manuscript review. Silvia Russel: data analysis and data interpretation, manuscript review. Hanneke WM van Laarhoven: study design, patient recruitment, data analysis and data interpretation, manuscript writing, manuscript review. Esther Helmich: study design, data analysis and data interpretation, manuscript writing, manuscript review.

## APPENDIX 1

**Table A1:** The characteristics of the patients (n = 18)

Participant number	Date interview	Sex	Age	Type of cancer	Months between diagnosis^a^ and interview	Does the patient receive palliative treatment?	Does the patient have other registered diagnoses?	WHO performance status
P1	Feb. 2018	Male	66	Esophagus	46	Yes	Yes	0
P2	Mar. 2018	Male	31	Esophagus	27	Not anymore	No	0
P3	Mar. 2018	Female	64	Esophagus	13	Yes	Yes	0
P4	Mar. 2018	Male	65	Esophagus	37	Yes	Yes	0
P5	Mar. 2018	Female	59	Pancreas	2	No	Yes	Unknown
P6	Mar. 2018	Male	49	Stomach	2	Yes	No	1
P7	Mar. 2018	Male	45	Stomach	33	Yes	No	0
P8	Mar. 2018	Female	68	Esophagus	4	Yes	Yes	1
P9	Mar. 2018	Female	63	Esophagus	27	Yes	Yes	2
P10	Mar. 2018	Female	56	Stomach	8	Yes	Yes	1
P11	Apr. 2018	Female	75	Ovary	19	Yes	Yes	1
P12	Apr. 2018	Female	46	Stomach	14	Yes	Yes	1
P13	Apr. 2018	Male	79	Colon	33	Yes	Yes	2
P14	Apr. 2018	Female	73	Pancreas	7	Yes	No	1
P15	Apr. 2018	Female	81	Esophagus	32	Yes	Yes	1
P16	Apr. 2018	Male	71	Esophagus	40	Yes	Yes	1
P17	Apr. 2018	Female	60	Pancreas	4	Yes	No	1
P18	May 2018	Female	73	Pancreas	21	Yes	Yes	0

a Specifically the diagnosis of advanced cancer.
